# RETRACTION: Clinical Effects of Acupuncture for the Treatment of Pressure Ulcers: A Comprehensive Systematic Review and Meta‐Analysis

**DOI:** 10.1111/iwj.70668

**Published:** 2025-04-23

## Abstract

**RETRACTION:** GuoY. , QiaoP. , ZhangK. , SunG. , WangR. , and YangX. , “Clinical Effects of Acupuncture for the Treatment of Pressure Ulcers: A Comprehensive Systematic Review and Meta‐Analysis,” International Wound Journal 21, no. 2 (2024): e14694, 10.1111/iwj.14694.PMC1082274842052915

The above article, published online on 28 January 2024, in Wiley Online Library (http://onlinelibrary.wiley.com/), has been retracted by agreement between the journal Editor in Chief, Professor Keith Harding; and John Wiley & Sons Ltd. Following an investigation by the publisher, all parties have concluded that this article was accepted solely on the basis of a compromised peer review process. The editors have therefore decided to retract the article. The authors did not respond to our notice regarding the retraction.

**RETRACTION:** Y. Guo , P. Qiao , K. Zhang , G. Sun , R. Wang , and X. Yang , “Clinical Effects of Acupuncture for the Treatment of Pressure Ulcers: A Comprehensive Systematic Review and Meta‐Analysis,” International Wound Journal 21, no. 2 (2024): e14694, 10.1111/iwj.14694.PMC1082274842052915

The above article, published online on 28 January 2024, in Wiley Online Library (http://onlinelibrary.wiley.com/), has been retracted by agreement between the journal Editor in Chief, Professor Keith Harding; and John Wiley & Sons Ltd. Following an investigation by the publisher, all parties have concluded that this article was accepted solely on the basis of a compromised peer review process. The editors have therefore decided to retract the article. The authors did not respond to our notice regarding the retraction.

